# A systematic review of psychosocial therapies for children with rheumatic diseases

**DOI:** 10.1186/s12969-016-0133-1

**Published:** 2017-01-17

**Authors:** Ezra M. Cohen, Alessio Morley-Fletcher, Darshan H. Mehta, Yvonne C. Lee

**Affiliations:** 1 Division of Immunology, Boston Children’s Hospital, 300 Longwood Ave, Boston, MA 02115 USA; 2 Department of Pediatrics, Massachusetts General Hospital, 55 Fruit Street, Boston, MA 02114 USA; 3 Benson-Henry Institute for Mind-Body Medicine, Massachusetts General Hospital, 151 Merrimac St., Boston, MA 02114 USA; 4 Division of Rheumatology, Immunology and Allergy, Brigham and Women’s Hospital, 60 Fenwood Road, Boston, MA 02115 USA; 5 Department of Rheumatology, Boston Children’s Hospital, 300 Longwood Ave, Boston, MA 02115 USA

**Keywords:** Juvenile arthritis, Juvenile fibromyalgia, Psychological therapies, Mind-body therapies, Meditation

## Abstract

**Background:**

To assess the quality of evidence for the effects of psychosocial therapies on pain and function in children with rheumatic diseases.

**Methods:**

We conducted a literature search of MEDLINE and PsycINFO for randomized clinical trials of psychosocial interventions for pain and disability in children with rheumatic diseases from January 1969 to September 2015. Studies with a sample size less than 10 subjects were excluded. Study quality was assessed using the Jadad score.

**Results:**

Five articles met inclusion criteria, for a total of 229 patients, aged 5 to 18 years. Two studies included children with fibromyalgia. Three studies included children with juvenile arthritis. Neither study in fibromyalgia reported the statistical significance of immediate between-group pre-post changes in functioning or pain. One study examining the effects of an internet-based psychosocial intervention in children with juvenile arthritis reported significant differences in post-intervention pain scores (*p* = 0.03). However, 2 studies did not show improvements in pain scores among children with juvenile arthritis treated with psychosocial interventions vs. a wait-list control or vs. an active control (massage). No studies reported significant between-group differences for functional outcomes in children with juvenile arthritis.

**Conclusions:**

The available data were limited by the scarcity of randomized trials. Definite conclusions about the immediate effect of psychosocial interventions on pain and function in children with fibromyalgia could not be made because between-group comparisons of post-treatment change scores were not reported. For children with juvenile inflammatory arthritis, results of between-group comparisons for pain differed across studies, and analyses examining disability revealed no significant differences between groups.

## Background

Children with rheumatic conditions often experience pain and disability [1, 2], leading to significant impairment in quality of life [3]. Pain and disability are frequently associated with mood disorders. One study noted that 67% of children with fibromyalgia have a current DSM-IV diagnosis [4]. Stress and mood problems may be associated with pain and disability [5, 6]. Given the association between pain, disability and psychosocial factors, psychosocial therapies have been hypothesized to have benefit for individuals with persistent pain and disability.

Psychosocial therapies may be defined as any therapies that influence the psychosocial processes which underpin or maintain pain, disability or other symptoms [7]. Among these, mind-body therapies and cognitive-behavioral therapies are the most widely used. Mind-body therapies are defined by the National Center for Complementary and Integrative Health as “a large and diverse group of techniques that are administered or taught to others by a trained practitioner or teacher [8].” Mind-body comprises the most commonly used complementary and alternative therapies, such as acupuncture, biofeedback, hypnosis, progressive muscle relaxation, guided imagery, mindfulness, meditation, yoga, tai chi and qi gong [9].

In the last few decades, cognitive-behavioral therapy (CBT) has been a dominant approach for treating psychological disorders in children, and though it shares many similarities with mind-body therapies, it is regarded by most as distinct [10]. CBT developed as a hybrid of cognitive and behavioral therapy [11]. Cognitive therapy focuses on identifying cognitive distortions and substituting them with more rational assessments. Behavioral therapy encourages behaviors that bring pleasure and self-efficacy, while discouraging maladaptive behaviors. Over time, CBT has absorbed techniques from a wide variety of interventions, including hypnosis, imagery, biofeedback, mindfulness techniques and meditation [12]. In fact, the term “cognitive-behavioral intervention” has become so broad that it may encompass its traditional meaning in addition to any or all of these techniques [7]. For this reason, it is difficult to estimate the effects of CBT in the literature as its own separate entity.

Psychosocial therapies have shown promise for treating rheumatic diseases in adults, though results are mixed. A large systematic review of psychological treatments in adults with rheumatoid arthritis found pooled effect sizes of *d* = 0.22 for pain and *d* = 0.27 for functional disability [13]. A systematic review of mind-body therapies for fibromyalgia found that individuals treated with mind-body therapies had greater improvements in pain and global assessment than wait-list controls, but poorer outcomes than individuals who participated in moderate/high intensity exercise [14]. In addition, a more recent randomized control trial (RCT) of tai chi vs. education and stretching in fibromyalgia concluded a modest benefit in symptoms [15].

Given studies reporting that psychosocial interventions improve pain and disability in adults with rheumatic conditions, we wanted to examine the evidence for psychosocial treatments to manage pain and disability in children with rheumatic disorders.

## Methods

### Inclusion and exclusion criteria

We included articles in this study only if they were published in English in a peer-reviewed journal. Unpublished abstracts and posters were excluded. We included articles which examined psychosocial therapies for children with rheumatic diseases with pain or disability as an outcome. Psychosocial therapies included CBT, coping skills, meditation, mindfulness, guided imagery, hypnosis, biofeedback, breathing techniques, progressive muscle relaxation, relaxation therapy, yoga and tai chi. Only prospective studies with a randomized design and total sample size > 10 were included in this review. We focused on prospective studies with a randomized design because we were interested in examining the impact of psychosocial therapies as an intervention. The study population included children who were 0 to 18 years at the beginning of the study. Articles which reported pain and functional disability as an outcome were included. Studies of children with any rheumatic disease (as below) were included.

Two investigators (EMC and AMF) independently conducted systematic searches for articles on psychosocial interventions in children through September 2015 on MEDLINE and PsycINFO using the following terms: (cognitive behavioral therapy, hypnosis, mindfulness, meditation, relaxation, progressive muscle relaxation, breathing, guided imagery, tai chi, or yoga) AND children AND rheumatic diseases (including fibromyalgia, systemic lupus erythematosus, juvenile arthritis, dermatomyositis, polymyositis, Sjogren’s, sarcoidosis, relapsing polychondritis, microscopic polyangiitis, granulomatosis with polyangiitis, eosinophilic granulomatosis with polyangiitis, polyarteritis nodosa, Takayasu arteritis, Behcet’s disease, and scleroderma). The two investigators compared search results, and discrepant results were re-reviewed and discussed until consensus was achieved.

The following data were extracted from articles for examination: population, country of origin, gender proportion, sample size, study design (randomized), type of control used if any, primary and secondary outcome measures, results, and duration of follow-up. When possible, we contacted the authors for information that was not provided in the manuscript. We calculated percentage change in pain and disability based upon pre and immediate post-treatment measures. “Post-treatment” refers to scoring just after the intervention finishes, and is a more proximate measure of the intervention’s effect, whereas end-of-study scores (i.e. after intervention-free follow-up) measure the durability of an intervention’s effects after it has been withdrawn. We assessed study quality using the Jadad score (0–5), a system that evaluates blinding, randomization and accounting for all subjects [16]. The Jadad score refers primarily to RCTs, as many of its components (blinding, randomization) are unique to RCTs.

## Results

**Fig. 1:**
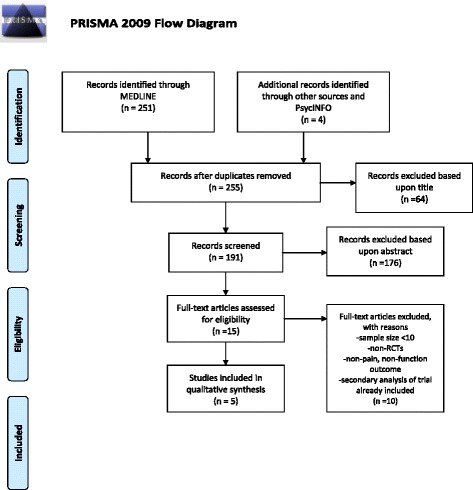
The process of study selection. “Other sources” include the references or text of reviewed articles

Our search yielded 255 manuscripts, of which 64 were excluded based on title because they did not involve children, rheumatic disease and/or psychosocial therapies. Of the remaining 191 manuscripts, 176 were excluded after reading the abstract because these studies included the wrong population, did not represent a prospective trial, and/or did not pertain to a childhood rheumatic disease. Fifteen manuscripts were read in full. Of those, 10 were excluded for *N* < 10, lacking randomization, analysis of previously reported results or lacking pain or function as an outcome [17]. This yielded 5 RCTs (Fig. 1). We divided these into trials of juvenile fibromyalgia and trials of juvenile arthritis because the pathophysiology of these conditions differs, with fibromyalgia being a non-inflammatory condition and juvenile arthritis being a systemic inflammatory condition. Tables 1 and 2 provide a summary of our results [18–22].

**Table 1:** RCTs in juvenile fibromyalgia

Citation	N	Age (years)	Gender (%F)	Study Design	Intervention	Comparison Group	Duration of Treatment	Additional Follow-up	Pain Measure	Pain results (post-treatment)	Disability Measure	Disability results (post-treatment)	Jadad Score
Kashikar-Zuck, S. et al. (2012) [22]	114	11–18	92	RCT	Weekly CBT sessions	Education group	8 weeks	4 months	Visual Analogue Scale (0–10). Higher score implies greater pain.	Pre-post difference in CBT group: 5.7 to 5.3 (−7%; *p*-value not provided)	Functional Disability Inventory (0–60)	Pre-post difference in CBT group: 21.4 to 16.7 (−22%; *p*-value not provided)	3
										Pre-post difference in education group: 5.8 to 6.0 (+3%; *p*-value not provided)		Pre-post difference in education group: 19.2 to 19.8 (+3%; *p*-value not provided)	
										Between groups difference of change scores: 0.6 (favoring CBT group; *p*-value not provided)		Between groups difference of change scores: 5.3 (favoring CBT group; *p*-value not provided)	
Kashikar-Zuck, S. et al. (2005) [21]	30	13–17	100	RCT (Crossover)	Weekly coping skills training	Self-monitoring (8 weeks of recording VAS scores)	8 weeks	8 weeks (to finish crossover intervention)	Visual Analogue Scale (0–10)	Pre-post difference in Group A (coping skills): 5.7 to 4.4 (−23%; *p*-value not provided)	Functional Disability Inventory (0–60)	Pre-post difference in Group A (coping skills): 21.0 to 15.1 (−28%) (*p*-values not provided)	3
										Pre-post difference in Group B (self-monitoring): 5.3 to 5.9 (+11%; *p*-value not provided)		Pre-post difference in Group B (self-monitoring): 21.9 to 16.6 (−24%) (*p*-values not provided)	
										Between group difference of change scores: 1.9 (favoring coping skills; *p*-value not provided)		Between group difference of change scores: mean 0.6 (favoring coping skills; *p*-value NS)	

The crossover design assigned patients to coping skills first then self-monitoring or vice versa

**Table 2:** RCTs in juvenile arthritis

Citation	N	Age (years)	Gender (%F)	Study Design	Intervention	Comparison Group	Duration of Treatment	Additional Follow-up	Pain Measure	Pain Results	Disability Measure	Disability Results	Jadad Score
Field T. et al. (1997) [18]	20	5–15	70	RCT	Relaxation therapy (nightly, parent-guided)	Daily massage	30 days	None	Visual Analogue Scale (0–10) (pain in the last week)	Pre-post difference in relaxation group: 3.5 to 4.2 (+20%; *p*-value NS)			1
										Pre-post difference in massage group: 4.8 to 1.6 (−67%; *p* < 0.05)			
										Between group difference of change scores: 3.9 (massage group with greater improvement; *p*-value not provided)			
Lomholt J. et al. (2015) [19]	19	9–14	79	RCT with group randomization	Weekly CBT sessions	Wait-list control	8 weeks	None	Revised Faces Pain Scale (0–10). Higher score implies greater pain.	Pre-post difference in CBT group: 3.1 to 4.1 (+32%; *p*-value NS)	Functional Disability Inventory (0–60)	Pre-post difference in CBT group: 11.4 to 11.7 (+2.6%; *p*-value NS)	2
										Pre-post difference in control group: 2.7 to 2.7 (0%; *p*-value NS)		Pre-post difference in control group: 9.8 to 9.2 (−6.1%; *p*-value NS)	
										Post-scores between groups adjusted for pre-scores: *p*-value NS		Between group differences in change scores: 0.9 (control group with greater improvement *p*-value NS)	
Stinson J. et al. (2010) [20]	46	12–18	67	RCT	Internet-based education, relaxation and distraction	Attention control	12 weeks	None	Recalled Pain Inventory (0–10) in past week. Higher score implies greater pain. [24]	Pre-post difference in intervention group; 2.7 to 2.2 (−19%; *p*-value not provided)	Juvenile Arthritis Quality of Life Questionnaire (JAQQ) activity (0–7; higher score implies greater dysfunction)	Pre-post difference in intervention group; 2.4 to 2.0 (−17%; *p*-value not provided)	2
										Pre-post difference in attention control 3.0 to 3.5 (+17%; *p*-value not provided)		Pre-post difference in attention control 2.7 to 2.3 (−15%; *p*-value not provided)	
										Post-score difference adjusted for pre-score significant (favoring intervention group; *p* = 0.03)		Post-score difference adjusted for pre-score: *p*-value NS	

We found a total of 2 studies for fibromyalgia, both from the same first author [21, 22]. These studies comprised a total sample size of 144 children. For mind-body interventions, 1 study used CBT and 1 used coping skills training. For controls, 1 study used education controls and 1 used self-monitoring, which involved symptom diaries. Treatment duration was 8 weeks for both studies. Follow-up ranged from 8 weeks to 6 months. Although there was significant drop-out (16 out of 114 subjects) in one study, there were no significant imbalances in the arms [22]. Both trials were RCTs, with a mean Jadad score 3. Both trials fully accounted for their participants and were single-blind. Neither were double-blind, underscoring the difficulty of double-blinding with psychosocial interventions. Both studies had weekly treatment sessions. Home practice was encouraged, but duration of home practice was not reported.

We found 3 studies in juvenile arthritis. These were from 3 different lead authors from 3 different countries, with a total sample size of 85 children. One study used progressive muscle relaxation as the primary intervention, while one used CBT, and one used computer-based CBT. For controls, one used an attention control (similar to self-monitoring), one used a wait-list control, and one used daily massage. 2 of the trials were RCTs, while the study by Lomholt et al. employed group randomization after stratifying by age and geographic region. These studies had a mean Jadad score of 1.6, and none were blinded. The median number of subjects in each trial was 20 with a range of 19 to 46. One had 9 subjects drop-out, with equal distribution of drop-outs between the intervention and control groups [20]. 1 of 19 subjects dropped out in the study by Lomholt et al., and drop-out was not reported in the study by Field et al. In all 3 studies, participants were followed for the duration of the interventions (4–12 weeks, median of 8 weeks), without additional follow-up beyond the intervention period. Home practice was a part of all studies’ treatment conditions, though mean duration of home practice was not reported.

The choice of pain and function outcomes were the same for the fibromyalgia studies, which all had the same first author, but varied across the studies of children with juvenile arthritis. The studies in fibromyalgia consistently used the Visual Analogue Scale (VAS; for pain) and Functional Disability Inventory (FDI; for disability). For the studies in arthritis, 1 study used the FDI while another used the Juvenile Arthritis Quality of Life Questionnaire (JAQQ), which is specifically designed to assess disability and dysfunction in children with arthritis [23]. The JAQQ assesses psychosocial, gross motor and fine motor functioning, as well as general symptoms. For pain, each of these studies used a different measure (e.g., VAS pain scale, Revised Faces Pain Scale and Recalled Pain Inventory) [24, 25].

In children with fibromyalgia, psychosocial therapies appeared to decrease disability, but documentation of statistically significant changes was frequently unavailable, particularly for immediate post-intervention scores. Percent improvement in FDI for the intervention groups ranged between 22 and 28%. In the comparison arms, improvement ranged from a worsening of 3% to an improvement of 24%. *P*-values for pre-post changes in FDI were not available for immediate post-intervention measures. Between group differences in the change in FDI score were non-significant in one study (comparing weekly coping skills training vs. self-monitoring) and not reported in the other.

For the outcome of pain among children with fibromyalgia, both studies reported VAS pain as an outcome and neither reported post-intervention significance levels. Between-group comparisons were not provided.

In children with juvenile arthritis, only two studies reported disability as an outcome measure. In these studies, between-group post-treatment disability scores did not significantly differ from each other. Pre-post significance levels were only provided in 1 of 2 studies, and this was not significant [19]. Stinson et al. reported a non-significant post-score difference (adjusted for pre-treatment baseline). This study used attention control with JAQQ as the outcome, while Lomholt et al. used wait-list control with FDI as the outcome.

For the outcome of pain in children with juvenile arthritis, no studies reported significant pre-post test results for the psychosocial intervention group. Between group comparisons of post-intervention pain scores revealed a statistically significant difference (*p* = 0.03), favoring the psychosocial intervention (internet-based education, relaxation and distraction) in one study [20], revealed no significant difference in one study [19] and were not reported in one study [18]. The latter study included an active control group (massage). Effect sizes could not be calculated because intra-class and inter-class correlations are required for these calculations, and these values were not reported in any of the studies. No adverse outcomes were reported in any of the studies.

## Discussion

To our knowledge, this is the first summary of the literature for psychosocial interventions for pediatric rheumatic disease. In children with fibromyalgia, within-group comparisons suggest that CBT/coping skills training may decrease pain and disability, but definite conclusions cannot be made because these studies did not report p-values for tests directly comparing pre-post-treatment change scores between groups. In studies of children with inflammatory arthritis, between-group comparisons did not reveal any significant differences in functional outcomes, and results of between-group comparisons for pain were not consistent across studies.

In children, psychosocial interventions have been studied most in behavioral and psychiatric conditions. One meta-analysis of mindfulness interventions in children found effect sizes in the small-to-moderate range, with larger effect sizes noted for improvements in psychosocial symptoms and for children treated in clinical settings rather than schools [26]. Yoga has been studied as an aid to physical therapy in rehabilitation, and other mind-body therapies have shown benefit for headache in children [27, 28]. Biofeedback also has a history of use in the pediatric population, treating conditions ranging from fecal and urinary incontinence to ADHD and headaches [29, 30]. CBT has been used most extensively in psychiatric conditions, notably anxiety and trauma [31, 32].

A prior systematic review and meta-analysis of psychosocial therapies in children with chronic pain suggests a large effect size, although only a subset of articles could be compared directly because they used a common pain measure [7]. Another Cochrane review evaluated psychosocial therapies for needle-associated procedural pain in children, finding large effect sizes for CBT, hypnosis and distraction [33]. However, many of the component studies in these reviews suffered from small sample size and heterogeneity of methods. In this review, the analyses examining pain as an outcome were less conclusive than those of Eccleston’s meta-analysis, which focused mainly on children with headaches [7]. Our study represents two smaller subpopulations (children with fibromyalgia and children with juvenile arthritis) which may respond differently to psychosocial therapies, as arthritis is thought to be primarily inflammatory in etiology, whereas fibromyalgia is driven primarily by central sensitization. One complicating feature of this distinction is that fibromyalgia can co-exist in children diagnosed with juvenile arthritis.

Our review was limited by the small number of appropriate studies, as well as the small sample size of the included studies. In addition, not all studies included both pain and disability as outcomes, limiting conclusions that could be made about both outcomes. Study quality was good in the fibromyalgia group (Jadad score of 3), though, due to the nature of the interventions, no studies were blinded. In the inflammatory arthritis group, study quality was poor (Jadad score of 1.6). Similar to the studies of children with fibromyalgia, these studies were not blinded, and many lacked adequate description of randomization methods. It should be noted that the applicability of the Jadad score has been questioned, given its emphasis on the reporting of methods rather than an evaluation of the methods themselves [16]. While there may be more consistency across the studies in the fibromyalgia, owed to their having a common first author, conclusions about their external validity are more difficult to draw. No studies in these groups reported adverse outcomes, which are likely rare with these therapies, but still essential to understand in order to assess risk-benefit ratios.

Other factors limit our ability to draw broad conclusions from these studies. In these studies, the duration of treatment ranged from 30 days to 12 weeks. It is unclear whether an intervention of this duration is long enough to have a measurable impact. We chose to assess the measurement of change in outcome immediately at the end of the intervention period because the greatest likelihood for improvement would be at this time point before the effects of the intervention fade. In future studies, it will be important to assess adherence to home therapies over time, as well as family and societal factors that may impact the effectiveness of psychosocial therapies.

In addition, there was significant heterogeneity in the length of treatment, making comparisons difficult. Though the studies were randomized, sample sizes were small and residual confounders, such as disease activity, may still be present. The generalizability of these results may also be limited because the study populations were mostly female adolescents. Since fibromyalgia and juvenile inflammatory arthritis affect females more them males, the female predominance in these studies is expected. Larger studies, however, with a larger number of males, are needed before the implications of these studies can be expanded to include boys and young men.

In addition, both studies of juvenile fibromyalgia and 1 study of juvenile arthritis excluded children less than 11 years of age [20–22]. This may reflect the perception that psychosocial therapies are easier to administer in older children, though CBT is used with regularity in children as young as 7 years old [34]. Whereas juvenile fibromyalgia disproportionately affects adolescents, juvenile arthritis generally peaks in the younger years [35, 36]. Additional studies are needed to understand whether the effectiveness of these interventions vary by age.

One of the greatest difficulties we encountered was the inconsistent reporting of outcomes and analyses. We chose to use the between-group difference in pre-post change scores as our primary outcome measure. These analyses directly compare changes in the outcome between the two groups, taking into account baseline values of the outcome. It is particularly important to consider baseline values of the outcome measure in small studies, where randomization may not yield groups with similar baseline characteristics. Although some studies reported a significant improvement in outcome measures in the treatment arm, adjustment for pre-post differences was frequently not reported.

Finally, while many studies used an education control, others used wait-list, self-monitoring and massage as comparison groups. To prove efficacy, it is important to know *over what*. This is an easier question to answer in drug trials, where placebo control groups have helped isolate drug effects from more nebulous causes. In research involving psychosocial interventions, which is the relevant comparison group-- standard medical care, wait-list controls, placebo interventions or other psychosocial interventions? In fact, all of these have been studied, with differing results. Interestingly, a large meta-analysis of mindfulness studies in adults by Grossman et al. showed similar mean effect sizes whether using active or wait-list controls [37]. Another large meta-analysis of CBT for adults with chronic pain by Morley et al. found considerable variability between wait-list and active controls, with wait-list controlled trials reporting larger differences in outcomes between the intervention and control groups [38].

## Conclusion

Nevertheless, many children with rheumatic conditions have pain that is incompletely treated, and there is a great demand for psychosocial and non-pharmacologic treatments. Based on this review, we conclude that the existing studies are too heterogeneous, and the reporting too inconsistent to draw convincing conclusions. In addition, although Eccleston’s earlier review suggested that adverse events, when reported, tend to be evenly distributed between control and intervention arms, there is a great need for consistent documentation of adverse effects in these studies, as these treatments are often thought of as being ‘risk-free’ when, in actuality, this area may just be ‘data-free’ [7]. The emergence and consolidation of research networks and registries in the pediatric rheumatology community such as the Childhood Arthritis and Rheumatology Research Alliance represent a major opportunity for studying these therapies in a uniform and broad-based manner, and these efforts will hopefully clarify the role of these therapies in the future [39].

## Abbreviations

CBT: Cognitive-behavioral therapy
FDI: Functional Disability Inventory
JAQQ: Juvenile Arthritis Quality of Life Questionnaire
RCT: Randomized controlled trial
VAS: Visual Analogue Scale.
